# Impacts of Substituting Fish Meal With Hydrolyzed Feather Meal on the Growth Performance, Immunity, and Antioxidant Capacity of Juvenile Largemouth Bass (*Micropterus salmoides*)

**DOI:** 10.1155/anu/6564426

**Published:** 2025-04-21

**Authors:** Yanhong Zhou, Hualiang Liang, Mingchun Ren, Dongyu Huang, Jiaze Gu

**Affiliations:** ^1^College of Fisheries and Life of Science, Shanghai Ocean University, Shanghai 201306, China; ^2^Key Laboratory of Integrated Rice-Fish Farming Ecology, Ministry of Agriculture and Rural Affairs, Freshwater Fisheries Research Center, Chinese Academy of Fishery Sciences, Wuxi 214081, China; ^3^Wuxi Fisheries College, Nanjing Agricultural University, Wuxi 214081, China

**Keywords:** antioxidant capacity, growth performance, hydrolyzed feather meal, immune function, juvenile largemouth bass

## Abstract

An 8-week breeding experiment was conducted to study the impacts of adding different levels (0%, 3.1%, 6.2%, 9.3%, 12.4%, and 15.5%) of hydrolyzed feather meal (HFM) in place of fish meal (FM) in the feed on the growth performance, immune function, and antioxidative ability of juvenile largemouth bass (*Micropterus salmoides*), with fishmeal substitution levels (FSLs) of 0% (control group; FSL0), 10% (FSL10), 20% (FSL20), 30% (FSL30), 40% (FSL40), and 50% (FSL50), respectively. The findings show that there were no notable differences observed among the different treatment groups when contrasted with FSL0. However, as the substitution level increased, final body weight (FBW), weight gain rate (WGR), and specific growth rate (SGR) declined, while the feedback coefficient rate (FCR), condition factor (CF), hepatosomatic index (HSI), and viscerosomatic index (VSI) increased. With an increase in the FSL, catalase (CAT) activity increased in all the groups and was significantly higher in the FSL20 and FSL50 groups than the control group; SOD activities in FSL40 and FSL50 significantly increased, and the plasma MDA contents in FSL40 and FSL50 significantly decreased. The gene expression levels of IL-10 and IL-8 in the groups FSL20 to FSL50 significantly decreased. When compared with *FSL0*, the gene expression levels of *CHOP* and *ATF6* were also significantly lower in the FSL10 to FSL50 groups. The overall expression level of *ASK1* was significantly reduced in the FSL20 group. Similarly, the expression level of *JNK1* was also significantly reduced in the FSL20 group. To sum up, replacing FM with HFM at 50% did not impact the growth of juvenile largemouth bass. An FSL range of 20%–50% can enhance the antioxidant capacity of largemouth bass, reduce inflammation and stress states, and have beneficial effects on the body. It is beneficial for maintaining the healthy growth of largemouth bass.

## 1. Introduction

Largemouth bass (*Micropterus salmoides*), a common carnivorous freshwater fish, is extensively cultivated in China because of its quick growth and soft meat [1]. The protein needs of largemouth bass typically range from 40% to 50% [2], with fish meal (FM) being considered the primary protein source in their feed [3]. However, due to the decline in fishery resources and the continuous increase in the price of FM [4], finding alternative protein sources to replace FM has become the key to the sustainable advancement of its aquaculture.

Currently, alternative protein sources that can meet the nutritional demands of aquatic animals mainly include plant-based and animal-derived proteins. Plant proteins are deficient in essential amino acids and minerals while containing high levels of anti-nutritional factors and cellulose [5]. In contrast, animal-derived proteins demonstrate superior characteristics as FM replacements due to their high protein content, balanced amino acid profile, abundant availability, and cost-effectiveness compared to FM [6]. Research has indicated that various animal protein sources can effectively substitute FM in the diet of largemouth bass. Specifically, chicken hemoglobin powder can replace at least 9.80% of FM [7], while fermented silkworm pupa powder can substitute 27%–30% of FM without adversely affecting growth, feed efficiency, or body shape index [8]. Additionally, enzymatically hydrolyzed intestinal mucosa protein can replace between 6.7% and 13.3% of FM in its diet [9]. Notably, spray-dried chicken plasma (SDCP) can substitute up to 57.14% of FM while enhancing antioxidant capacity [10]. Cricket meal has been shown to replace 45% of FM in juvenile largemouth bass feed without compromising specific growth rate (SGR) and feedback coefficient rate (FCR), while simultaneously increasing plasma superoxide dismutase (SOD) and catalase (CAT) activities [11]. Yellow mealworm (*Tenebrio molitor*) meal (YWM) can replace 25% of FM without growth performance impairment. However, substitution exceeding 50% negatively impacts both growth performance and hepatic health in largemouth bass [12]. These studies provide a useful reference for the optimization of largemouth bass feed formulations.

Feather meal, as an animal protein source, is processed from the feathers and down feather stalks of livestock and poultry. Its main component is the structural protein keratin [13]. However, untreated feather meal presents problems such as poor digestibility and low nutritional value [14, 15]. Therefore, feathers need to be hydrolyzed to partially degrade the keratin filaments to improve their absorption and digestibility [16, 17]. Hydrolyzed feather meal (HFM) is produced through the hydrothermal processing of feather meal [18]. It is an economical (usually 200–600 euros/ton) feed material rich in crude protein (usually 73%–96%) [19]. Because of its widespread availability and high protein levels, it can serve as a cost-effective alternative protein source in animal feed. Previous studies have shown that HFM can replace FM in fish feed to a certain extent for aquatic animals such as juvenile catfish (*Ictalurus punctatus*) [20], gilthead seabream (*Sparus aurata*) [19] and Pengze crucian carp (*Carassius auratus var. Pengze*) [21] to meet their protein requirements. However, there is limited research on how replacing FM with HFM impacts the growth, immune responses, and antioxidant properties of juvenile largemouth bass. Therefore, this research sought to explore the implications of replacing FM with HFM on these specific physiological aspects in largemouth bass. It is expected to provide a theoretical basis and practical guidance for the rational utilization of HFM in the feed of largemouth bass.

## 2. Materials and Methods

### 2.1. Design of Experiments

The diets were created following the principles of iso-nitrogen and iso-energy, with FM, soybean meal, and soy protein concentrate as the primary protein sources, while fish oil served as the main source of lipid. HFM was added at inclusion levels of 0% (control), 3.1%, 6.2%, 9.3%, 12.4%, and 15.5% to replace FM in the diets, corresponding to fishmeal substitution levels (FSLs) of 0% (FSL0), 10% (FSL10), 20% (FSL20), 30% (FSL30), 40% (FSL40), and 50% (FSL50), respectively. The experimental diets' formulas and chemical compositions are presented in Table 1. All the raw materials were procured from Biomar Tongwei Biotech Co., Ltd. (Wuxi, China). The materials underwent grinding to achieve a fine powder consistency, followed by sieving through an 80-mesh filter. The powder was then processed through a complete floating feed production line to form 1.5 mm pellets. Subsequently, the pellets were transferred to a drying oven. After drying, the material was elevated into a vacuum coating system for oil spraying to achieve lipid supplementation. The coated pellets then entered a cooling tower for temperature reduction. Then, all the feeds were stored in sealed bags and transferred to a −20 °C refrigerator for future use.

### 2.2. The Breeding and Management of Experimental Fish

Juvenile largemouth bass were sourced from the Freshwater Fisheries Research Center at the Chinese Academy of Fishery Sciences. After acclimation in floating cages for 2 weeks, the breeding experiment was conducted. After fasting, the fish for 24 h, 360 healthy juvenile fish, each with an average initial weight of 3.27 ± 0.07 g, were chosen and randomly distributed into 18 floating cages (1 × 1 × 1 m), with 20 fish in each cage, for the pond breeding experiment. A total of 18 floating cages were organized into 6 groups, each comprising 3 replicates. The experiment lasts for 8 weeks, with the weight of the fish recorded every 2 weeks. The fish are fed to satiation twice daily at 07:00 and 17:00 (during feeding, if the fish stop eating, feeding is immediately discontinued). After the experiment concludes, the total amount of feed used throughout the entire experiment is calculated. Throughout the experimental period, water temperature was maintained at 27 °C–30 °C, dissolved oxygen concentration remained ≥6 mg/L, and pH levels were stabilized between 7.0 and 7.8.

### 2.3. Sample Collection and Processing

After the breeding period ended, the fish were subjected to a fasting period of 24 h. Subsequently, the quantity of fish remaining in each floating cage, along with the total weight of the experimental subjects, was recorded. These data were utilized to compute various growth performance metrics, including the final body weight (FBW), weight gain rate (WGR), FCR, SGR, hepatosomatic index (HSI), viscerosomatic index (VSI), and condition factor (CF). Three fish were randomly selected from each cage, followed by the measurement of their body weights and body lengths. Then, blood samples were obtained from the caudal vein. After blood collection, the fish underwent dissection, during which the weights of the liver and visceral organs were recorded. Intestinal tissues of the fish were gathered and placed in pre-labeled cryopreservation tubes. These samples were briefly preserved in a fluid nitrogen environment and subsequently maintained at −80 °C for future analysis. The blood samples underwent centrifugation at 3000 rpm for 10 min and kept at 4 °C. Then, the supernatant was transferred to −80 °C for preservation to determine the blood biochemical indicators. Two fish were collected from each cage and preserved in a −20 °C freezer for the determination of entire fish body components.

### 2.4. Methods of Experimental Determination

The feed and entire fish body ingredients were subjected to analysis in accordance with the methodologies established by the AOAC [22]. The plasma alanine aminotransferase (ALT), aspartate aminotransferase (AST), total cholesterol (TC), triglyceride (TG), and glucose (GLU) were quantified utilizing a Mindray BS-400 automatic biochemical analyzer (Shenzhen, China). Plasma antioxidant parameters, including CAT, SOD, malondialdehyde (MDA), and total antioxidant capacity (T-AOC), were assessed using detection kits procured from the Jiancheng Bioengineering Institute (Nanjing, China).

The determination of relative mRNA expression involved several key steps, including the extraction of total RNA from tissue samples, the evaluation of the RNA concentration and quality, and real-time PCR analysis. A one-step quantitative PCR kit utilizing SYBR Green (Nanjing Vazyme Biotechnology Co., Ltd., Nanjing, China) was employed to quantify the mRNA levels of the target genes utilizing a CFX96 Touch (Bio-Rad, Hercules, CA, USA).  Table 2 details the specific primers utilized in this experiment. *GAPDH* served as the internal reference gene. The expression levels of the target genes were analyzed using the relative standard curve method.

### 2.5. Data Analysis

The experimental data were subjected to statistical analysis utilizing one-way analysis of variance, implemented through SPSS version 26.0. Before conducting the statistical analysis, it was confirmed that all the experimental data adhered to the assumptions of the homogeneity of the variance and a normal distribution. The results were presented as means ± SEMs. A *p*  < 0.05 was deemed statistically significant.

## 3. Results

### 3.1. Growth Performance

As indicated in Table 3, there were no statistically significant differences in the FBW, WGR, SGR, FCR, HSI, VSI, and CF of largemouth bass among the various treatment groups when compared to FSL0 (*p*  > 0.05).

### 3.2. Entire Fish Body Components

The composition of the entire fish body is presented in Table 4. The incorporation of different amounts of HFM into the diet did not produce a statistically significant impact on the moisture, crude protein, crude lipid, and ash contents of the entire fish (*p*  < 0.05).

### 3.3. Plasma Biochemical Indices

As shown in Table 5, compared with FSL0, a notable decrease in TC and TG was observed in FSL50 (*p*  < 0.05). However, no remarkable disparities in ALT and GLU levels were observed in the FSL10, FSL20, FSL30, FSL40, and FSL50 when compared to the FSL0 (*p*  > 0.05).

### 3.4. Plasma Antioxidative Indicators

**Figure 1:**
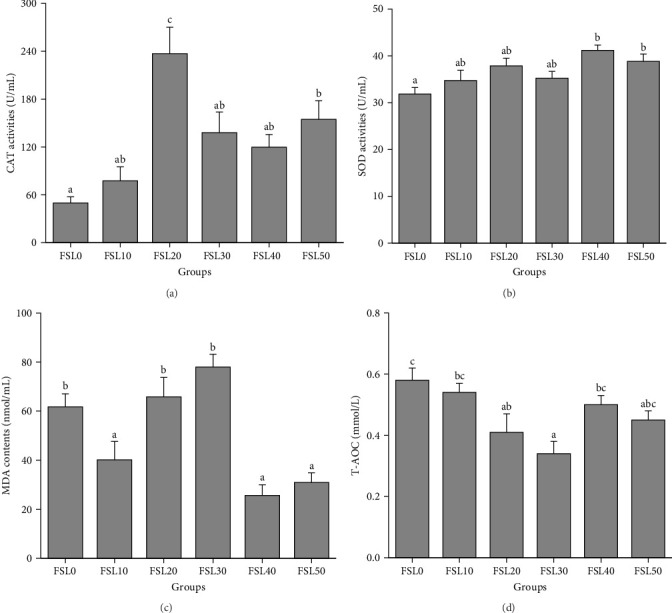
The effects of different fishmeal substitution level on the plasma antioxidant indicators of largemouth bass antioxidant-related parameters. (A) CAT; (B) SOD; (C) MDA; (D) T-AOC. Data were expressed as means with SEM. Distinct superscripts on the columns denote statistically significant differences (*p*  < 0.05).

As shown in Figure 1, with an increase in the FSL, the CAT activity was typically greater than that of FSL0, with FSL20 and FSL50 showing a significant increase compared to FSL0 (*p*  < 0.05; Figure 1A). Compared with FSL0, the SOD activity of FSL40 and FSL50 showed a marked increase (*p*  < 0.05; Figure 1B). The levels of MDA in FSL40 and FSL50 were significantly decreased compared with those in FSL0 (*p*  < 0.05; Figure 1C). Compared with FSL0, the T-AOC showed a downward trend, with FSL30 demonstrating a statistically significant difference (*p*  < 0.05; Figure 1D).

### 3.5. Intestinal Gene Expression

**Figure 2:**
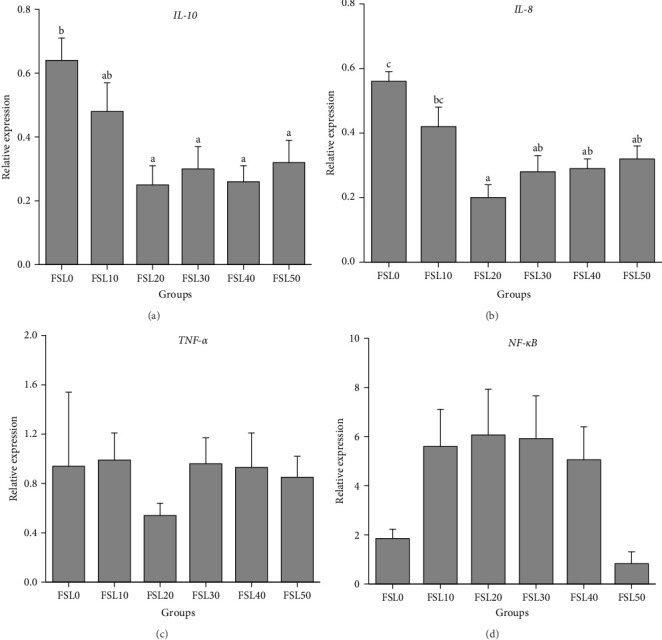
The effects of different fishmeal substitution level on gene expressions associated with intestinal immune inflammation in largemouth bass. (A) Interleukin 10 (IL-10); (B) interleukin 8 (IL-8); (C) tumor necrosis factor-*α* (TNF-*α*); (D) nuclear factor-kappa B (NF-*κ*B). Data were expressed as means with SEM. Distinct superscripts on the columns denote statistically significant differences (*p*  < 0.05).

As shown in Figure 2, compared with FSL0, the gene expression levels of IL-10 and IL-8 in the groups with added HFM showed a downward trend. Notably, the levels of mRNA expression in the FSL20, FSL30, FSL40, and FSL50 groups were significantly reduced (*p*  < 0.05; Figure 2A,B). Conversely, the mRNA levels of TNF-*α* and NF-*κ*B did not show significant differences when compared to FSL0 (*p*  > 0.05; Figure 2C,D).

**Figure 3:**
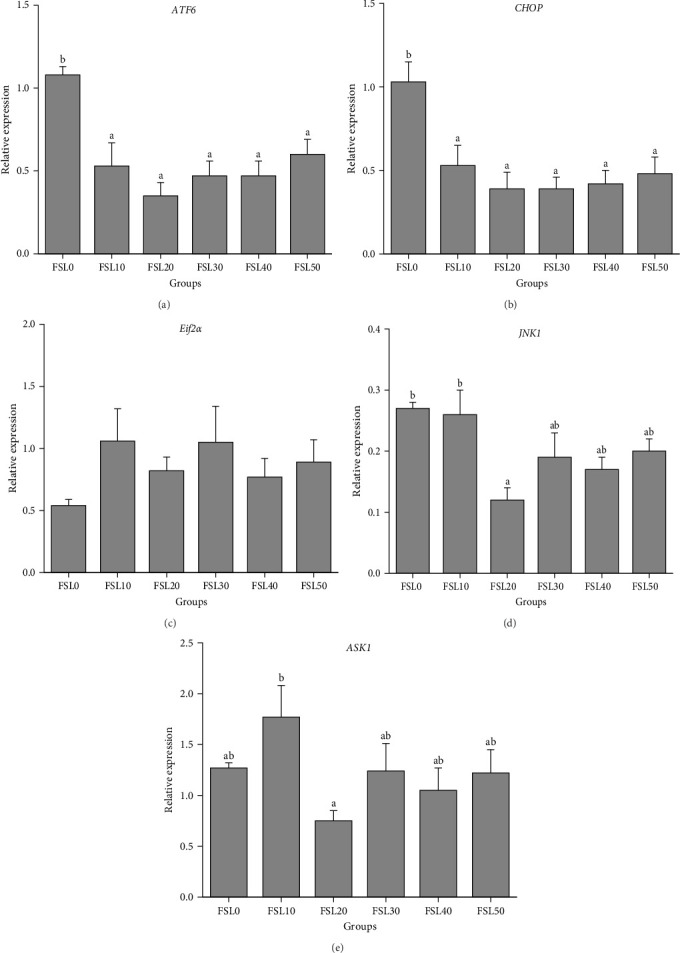
The impact of various fishmeal substitution level on the expressions of intestinal stress-related genes in largemouth bass. (A) Activating transcription factor 6 (ATF6); (B) C/EBP homologous protein (CHOP); (C) eukaryotic initiation factor 2*α* (Eif2*α*); (D) c-Jun N-terminal kinase 1 (JNK1); (E) apoptosis signal-regulating kinase 1 (ASK1). Data were expressed as means with SEM. Distinct superscripts on the columns denote statistically significant differences (*p*  < 0.05).

As shown in Figure 3, an increase in the FSL resulted in a significant reduction in the gene expression levels of *ATF6* and *CHOP* (*p*  < 0.05; Figure 3A,B). Conversely, different FSLs did not exert a significant impact on the gene expression of *Eif2*α (*p*  > 0.05; Figure 3C). When comparing FSL0 to FSL20, there was a notable decrease in the relative expression levels of the *JNK1* and *ASK1* genes (*p*  < 0.05; Figure 3D, E).

## 4. Discussion

Under the conditions of this experiment, with an increase in the FSL, the addition of HFM to substitute FM did not have a significant direct effect on growth, which was supported by the results for FW, WGR, SGR, and FCR. Hence, HFM might be highly acceptable for largemouth bass, being able to provide the nutrients necessary for their growth. Feather protein is abundant in a variety of amino acids, including glycine, proline, and hydroxyproline [27]. After hydrolysis, the structure of the protein changes, making it more easily digested and absorbed [28], thereby promoting the growth of largemouth bass. The research results regarding HFM in aquatic animals vary. Replacing 76% of the FM with HFM in the diet of juvenile European seabass (*Dicentrarchus labrax*) did not compromise its feed intake or growth performance [29]. HFM can replace up to 15% of the soybean meal (SBM) without affecting the growth performance of juvenile catfish [20]. Replacing 15%–45% of the FM with HFM also does not adversely impact the growth performance of Pengze crucian carp and can also improve the muscle quality of Pengze crucian carp [21]. Furthermore, it is viable to replace 33%–67% of the FM with HFM in river prawns' feed without affecting their growth performance [30]. However, when too much HFM is added, the growth performance indicators show a downward trend. Replacing 50% of the FM with HFM leads to a reduction in both the growth and feed efficiency of gilthead seabream, and the complete substitution of FM results in significantly worse fish performance [19]. Compared with FM, HFM contains lower levels of lysine, methionine, and other essential amino acids [31]. Therefore, a high substitution level of HFM cannot meet the fish's demand for essential amino acids, thereby affecting growth performance. These results suggest that there is a need to evaluate and develop quasi-definitive HFM use and fishmeal alternatives. Furthermore, different amounts of HFM in the diet did not produce a statistically significant impact on the overall body composition of the fish, encompassing parameters such as moisture, crude protein, crude lipid, and ash content, which was similar to the results for European sea bass [29]. At the same time, HFM has been shown not to significantly affect the nutrient contents of other fish [32–34].

Plasma biochemical parameters are important indicators reflecting the nutritional metabolism and health status of fish [35]. In this experiment, when the FSL reached 50%, TC and TG were significantly decreased, which might imply that the addition of HFM positively regulates lipid metabolism in fish. In Pengze crucian carp, after HFM was used to replace part of the FM, plasma parameters such as TG and TC were also significantly reduced [21]. Additionally, replacing 20% of the FM with HFM resulted in decreased cholesterol levels in Atlantic salmon (*Salmo salar* L.) [36]. Feather meal primarily consists of keratin and lacks cholesterol. Even after hydrolyzed partial degradation, its cholesterol content remains insufficient [13, 21]. Therefore, the significant reduction of plasma TC and TG content caused by the addition of HFM in the diet in this study is likely to be related to the deficiency of cholesterol in feather meal. The addition of HFM did not significantly influence the levels of GLU, ALT, and AST, which indicated that the partial substitution of FM with HFM did not damage the hepatic tissue of the fish. These findings are consistent with those reported in prior research. The replacement of 50% of the FM with HFM does not affect the hematological parameters of gilthead seabream [19].

The antioxidant performance of fish is an important indicator of their growth and health status [37]. In this experiment, with an increase in the FSL, the CAT activity exhibited an initial rise followed by a subsequent decline, with the peak value observed at FSL20. Compared with that in FSL0, the SOD activity of FSL40 and FSL50 increased significantly. However, compared with that in FSL0, the T-AOC showed a downward trend, and that in FSL30 showed a significant difference. Although the CAT activity and SOD activity increased at some addition levels, the decrease in T-AOC indicates that the incorporation of HFM may not comprehensively improve the overall antioxidant capacity of largemouth bass. This may be because a high addition level affects the metabolism or function of other antioxidant substances, or, although the activity of certain antioxidant enzymes is enhanced, other antioxidant mechanisms do not increase accordingly, resulting in an overall antioxidant effect that does not meet expectations [38, 39]. The specific reasons and regulatory mechanisms need to be further studied. MDA is an important by-product of lipid peroxidation and acts as a vital indicator of oxidative stress, reflecting tissue damage resulting from peroxidative processes [40]. In this experiment, the MDA content in FSL40 and FSL50 was significantly lower than that in FSL0. The reduction in MDA content means that the degree of lipid peroxidation in largemouth bass was reduced and the oxidative damage to cells was reduced, further supporting the idea that replacing FM with HFM could improve antioxidant capacity to some extent. Similar results have been found in Pengze crucian carp [21].

The inflammatory response is an important marker of the health of aquatic organisms and is integral to the immune system's reaction to foreign antigens, primarily regulated by cytokines [35, 41]. The activation of NF-*κ*B can induce structural damage to intestinal tissues by enhancing the expression of proinflammatory cytokines like TNF-*α* and IL-8, while also reducing the expression of anti-inflammatory cytokines, including IL-10 [42, 43]. In this experiment, after adding HFM to replace FM, the intestinal expression levels of NF-*κ*B and TNF-*α* were not statistically significantly different from those observed in FSL0. In addition, after adding HFM, the gene expression levels of intestinal IL-10 and IL-8 showed a downward trend, and the expression levels of these two mRNAs in FSL20 to FSL50 were significantly reduced. This indicated that replacing FM with HFM might have a certain inhibitory effect on inflammation. It should be noted that, after adding HFM, the mRNA abundances of IL-10 and IL-8 were decreased simultaneously. This might mean that, under the action of HFM, there is a coordinated linkage between anti-inflammatory factors and proinflammatory factors to jointly maintain the immune homeostasis of the body [44]. The reduction in proinflammatory factors suggests a reduction in the inflammatory response of the body and a healthier state. This may feedback and regulate the expression of anti-inflammatory factors, causing them to also decrease correspondingly [45]. Some studies have pointed out that anti-inflammatory cytokines can limit the production of proinflammatory cytokines [46]. When proinflammatory factors decrease, anti-inflammatory factors are also adjusted accordingly to maintain the body's immune balance [45]. In addition, endoplasmic reticulum stress has multiple roles in organisms. It is involved in antioxidant, immune, and apoptotic processes [47]. It mediates multiple pathways in cell apoptosis, among which *CHOP* is a key component [48]. In different studies, it has been found that *CHOP* is mainly activated through the *PERK-eIF2α-ATF4* pathway [49] and the *IRE1-TRAF2-ASK-JNK* pathway [29]. These pathways may play different roles under different physiological and pathological conditions and jointly regulate the apoptotic processes of cells. With an increase in the FSL, there was a notable reduction in the expression levels of the genes *CHOP* and *ATF6*, which exhibited comparable patterns. Compared with those in FSL0, the relative expression levels of the *ASK1* and *JNK1* genes in FSL20 decreased significantly. *ASK1* and *JNK1* are key genes in the *IRE1-TRAF2-ASK-JNK* apoptotic pathway [29, 50]. Under normal physiological conditions, endoplasmic reticulum stress can activate *CHOP* and then activate the *IRE1-TRAF2-ASK-JNK* signaling pathway to induce cell apoptosis [29]. After adding HFM, the expression levels of the *ASK1* and *JNK1* genes decreased, which may mean that the activity of this apoptotic pathway is reduced, thereby reducing the occurrence of cell apoptosis and being beneficial to the health of largemouth bass. This further supports the positive role of HFM in regulating intestinal gene expression, improving the body's immunity and stress state.

## 5. Conclusion

Overall, replacing 50% of FM in the diet with HFM has no effect on the growth performance of largemouth bass. In terms of antioxidant and immune capacity, the addition of HFM could also improve the antioxidant capacity to some extent and inhibit the inflammatory response of largemouth bass.

## Figures and Tables

**Table 1:** Formulation and proximate composition of experimental diets (dry matter basis, g/kg).

Ingredients	Diet					
	FSL0	FSL10	FSL20	FSL30	FSL40	FSL50
Fish meal^a^	45.00	40.50	36.00	31.50	27.00	22.50
Hydrolyzed feather meal^a^	0.00	3.10	6.20	9.30	12.40	15.50
Soy concentrated protein^a^	5.00	5.00	5.00	5.00	5.00	5.00
Soybean meal^a^	6.00	6.00	6.00	6.00	6.00	6.00
Blood meal^a^	5.00	5.00	5.00	5.00	5.00	5.00
Wheat meal	5.00	5.00	5.00	5.00	5.00	5.00
Chicken meal^a^	8.00	8.00	8.00	8.00	8.00	8.00
Gluten^a^	3.00	3.00	3.00	3.00	3.00	3.00
Fish oil	5.00	5.15	5.30	5.45	5.60	5.75
Cassava starch	5.00	5.00	5.00	5.00	5.00	5.00
Choline chloride	0.50	0.50	0.50	0.50	0.50	0.50
Vitamin premix^b^	1.00	1.00	1.00	1.00	1.00	1.00
Mineral premix^b^	1.00	1.00	1.00	1.00	1.00	1.00
Monocalcium phosphate	2.20	2.60	3.00	3.40	3.80	4.20
Rice bran	6.25	6.88	7.52	8.16	8.80	9.43
Vitamin C	0.05	0.05	0.05	0.05	0.05	0.05
L-lysine	0.00	0.16	0.31	0.47	0.62	0.78
L-methionine	0.00	0.06	0.12	0.17	0.23	0.29
Bentonite	2.00	2.00	2.00	2.00	2.00	2.00
Proximate composition						
Crude protein (%)	48.57	48.86	48.53	48.87	48.80	48.98
Crude lipid (%)	8.40	7.74	8.28	8.11	7.70	8.01
Gross energy (KJ/g)	17.82	17.83	17.84	17.83	17.85	17.86
Ca (%)	2.76	2.64	2.51	2.39	2.26	2.13
P (%)	1.93	1.95	1.92	1.94	1.95	1.96

^a^The levels of crude protein in various feed ingredients were as follows: fish meal at 65.80%, hydrolyzed feather meal at 85.10%, soy concentrated protein at 63.00%, blood meal at 90.00%, soybean meal at 46.00%, chicken meal at 62.00%, and gluten at 80.00%. In terms of crude lipid content, the values were 9.50% for fish meal, 2.50% for hydrolyzed feather meal, 4.10% for soy concentrated protein, 0.00% for blood meal, 4.25% for soybean meal, 9.00% for chicken meal, and 2.00% for gluten. The amino acid contents of hydrolyzed feather meal were arginine 6.06%, histidine 1.01%, isoleucine 4.21%, leucine 7.03%, lysine 2.16%, methionine 0.75%, phenylalanine 4.16%, threonine 3.97%, tryptophan 0.66%, valine 6.82%, alanine 4.09%, aspartic acid 6.21%, cysteine 3.81%, glutamic acid 7.11%, glycine 6.23%, proline 9.16%, serine 7.33%, and tyrosine 2.91%. The ingredients were sourced from Biomar Tongwei Biotech Co., Ltd. (Wuxi, China).

^b^The vitamin and mineral premixes were supplied by Hanove Animal Health Products Co., Ltd. (Wuxi, China).

**Table 2:** Real-time PCR primer sequences.

Gene	Forward sequence	Reverse sequence	Accession number/reference
*GAPDH*	ACTGTCACTCCTCCATCTT	CACGGTTGCTGTATCCAA	AZA04761.1
IL-10	CGGCACAGAAATCCCAGAGC	CAGCAGGCTCACAAAATAAACATCT	[23]
IL-8	CGTTGAACAGACTGGGAGAGATG	AGTGGGATGGCTTCATTATCTTGT	[23]
TNF-*α*	CTTCGTCTACAGCCAGGCATCG	TTTGGCACACCGACCTCACC	[24]
NF-*κ*B	CCACTCAGGTGTTGGAGCTT	TCCAGAGCACGACACACTTC	Cluster-21914.7253
*CHOP*	TGTGTTGTGTTTGCTTCGCC	AAAACGTTTGGCACGCTTCA	[25]
*ATF6*	GACGCCCCGCATAAGAGTAA	GCAGACTTGAGGAGAGCTGG	[26]
*Eif2ɑ*	CCTCGTTTGTCCGTCTGTATC	GCTGACTCTGTCGGCCTTG	[25]
*ASK1*	CAACTACGCCTTCATCCCGT	GGTCCCAACAGCATCTCGAA	[26]
*JNK1*	TGCACTACCTGAGCCACTTG	TGTGCTTCCTGGCTGATGTT	XM_038735152.1

**Table 3:** The effects of different FSL on the growth performance of largemouth bass.

Parameters	FSL0	FSL10	FSL20	FSL30	FSL40	FSL50
IBW (g)	3.29 ± 0.02	3.25 ± 0.02	3.24 ± 0.02	3.26 ± 0.00	3.28 ± 0.02	3.31 ± 0.02
FBW (g)	27.28 ± 2.00	27.20 ± 1.55	26.58 ± 3.04	23.23 ± 1.88	23.05 ± 0.34	24.67 ± 1.87
WGR (%)	729.22 ± 66.77	735.24 ± 42.54	718.61 ± 88.42	612.38 ± 56.70	603.59 ± 14.46	645.41 ± 59.75
SGR (%/day)	3.77 ± 0.14	3.79 ± 0.09	3.73 ± 0.18	3.50 ± 0.14	3.48 ± 0.04	3.58 ± 0.15
FCR	1.26 ± 0.06	1.29 ± 0.07	1.28 ± 0.02	1.39 ± 0.04	1.38 ± 0.06	1.39 ± 0.08
HSI (%)	2.36 ± 0.29	2.42 ± 0.39	2.41 ± 0.26	2.51 ± 0.22	2.41 ± 0.12	2.37 ± 0.22
VSI (%)	10.83 ± 0.37	11.35 ± 0.24	11.34 ± 0.62	11.23 ± 0.38	11.42 ± 0.30	12.02 ± 0.37
CF (g/cm^3^)	1.99 ± 0.05	2.10 ± 0.05	2.02 ± 0.06	2.00 ± 0.03	2.01 ± 0.03	2.14 ± 0.02

*Note:* Data presented in the form of mean ± SEM, different letters represent significant differences (*p*  < 0.05). Initial body weight (IBW); final body weight (FBW); WGR (%) = 100 × (final weight (g) − initial weight (g))/initial weight (g); SGR (%/day) = 100 × ((Ln (final body weight (g)) − Ln (initial body weight (g)))/days); FCR = dry feed fed (g)/(final body weight (g) − initial body weight (g)); CF (g/cm^3^) = 100 × fish weight (g)/(body length (cm))^3^; HSI (%) = 100 × (liver weigh (g)/body weight (g)); VSI (%) = 100 × (visceral weight (g)/body weight (g)).

**Table 4:** The effects of different fishmeal substitution level (FSL) on the whole-fish body composition of largemouth bass.

Parameters	FSL0	FSL10	FSL20	FSL30	FSL40	FSL50
Moisture (%)	71.92 ± 0.40	72.41 ± 0.34	72.23 ± 0.21	71.73 ± 0.40	72.93 ± 0.25	72.24 ± 0.58
Crude lipid (%)	6.03 ± 0.40	5.40 ± 0.38	6.05 ± 0.35	5.41 ± 0.33	4.83 ± 0.39	4.99 ± 0.60
Crude protein (%)	16.66 ± 0.12	16.75 ± 0.13	16.53 ± 0.34	17.26 ± 0.17	16.78 ± 0.32	16.93 ± 0.23
Ash (%)	3.97 ± 0.08	4.16 ± 0.15	3.97 ± 0.05	3.61 ± 0.30	4.07 ± 0.02	3.89 ± 0.10

**Table 5:** The effects of different fishmeal substitution level (FSL) on the plasma biochemical indicators of largemouth bass.

Parameters	FSL0	FSL10	FSL20	FSL30	FSL40	FSL50
ALT (U/L)	1.58 ± 0.54	2.20 ± 0.41	1.80 ± 0.20	1.45 ± 0.41	3.20 ± 0.54	2.88 ± 0.26
AST (U/L)	18.51 ± 1.41^ab^	19.11 ± 1.57^b^	16.21 ± 1.70^ab^	13.04 ± 1.53^ab^	14.41 ± 1.35^ab^	12.42 ± 1.58^a^
TC (mmol/L)	7.34 ± 0.31^c^	6.43 ± 0.11^bc^	6.59 ± 0.17^bc^	7.14 ± 0.28^c^	6.03 ± 0.24^ab^	5.34 ± 0.25^a^
TG (mmol/L)	9.99 ± 0.63^c^	8.24 ± 0.27^abc^	8.80 ± 0.54^bc^	10.06 ± 0.60^c^	7.51 ± 0.36^ab^	6.55 ± 0.39^a^
GLU (mmol/L)	10.15 ± 1.12	12.64 ± 1.03	11.62 ± 0.74	9.41 ± 0.71	9.98 ± 0.65	11.13 ± 0.88

*Note:* Data presented in the form of mean ± SEM, with different letters indicating statistically significant difference (*p* < 0.05).

## Data Availability

The authors confirm that the data supporting the findings of this study are available within the manuscript, Tables and Figures. Data are available from the corresponding author upon reasonable request.

## References

[1] He M., Li X., Poolsawat L. (2020). Effects of Fish Meal Replaced by Fermented Soybean Meal on Growth Performance, Intestinal Histology and Microbiota of Largemouth Bass (*Micropterus salmoides*). *Aquaculture Nutrition*.

[2] Huang D., Wu Y., Lin Y. (2017). Dietary Protein and Lipid Requirements for Juvenile Largemouth Bass, *Micropterus salmoides*. *Journal of the World Aquaculture Society*.

[3] Liu Y., Lu Q., Xi L. (2021). Effects of Replacement of Dietary Fishmeal by Cottonseed Protein Concentrate on Growth Performance, Liver Health, and Intestinal Histology of Largemouth Bass (*Micropterus salmoides*). *Frontiers in Physiology*.

[4] Food and Agriculture Organization of the United Nations (2016).

[5] Huang Y. J., Zhang N. N., Fan W. J. (2018). Soybean and Cottonseed Meals Are Good Candidates for Fishmeal Replacement in the Diet of Juvenile *Macrobrachium nipponense*. *Aquaculture International*.

[6] Donadelli R. A., Aguilar F. A., Sonoda D. Y., Cyrino J. E. P. (2019). Poultry by-Product Meal as Dietary Protein Source for Dourado, *Salminus brasiliensis*. *An Economic Appraisal, Scientia Agricola*.

[7] Ding G., Li S., Wang A., Chen N. (2020). Effect of Chicken Haemoglobin Powder on Growth, Feed Utilization, Immunity and Haematological Index of Largemouth Bass (*Micropterus salmoides*). *Aquaculture and Fisheries*.

[8] Zhang Q., Bian Y., Zhao Y. (2022). Replacement of Fishmeal by Fermented Silkworm Pupae Meal in Diets of Largemouth Bass (*Micropterus salmoides*). *Effects on Growth Performance and Feed Utilization, Journal of Applied Ichthyology*.

[9] Yu H., Ge X., Zhang L., Chen X., Ren M., Liang H. (2023). Transcriptome Analysis Reveals the Feeding Response and Oxidative Stress in Juvenile, *Micropterus salmoides*, Fed a Low-Fish-Meal Diet With Enzyme-Hydrolysed Intestinal Mucosa Protein Substitution. *Aquaculture*.

[10] Xu J., Sheng Z., Chen N., Xie R., Zhang H., Li S. (2022). Effect of Dietary Fish Meal Replacement With Spray Dried Chicken Plasma on Growth, Feed Utilization and Antioxidant Capacity of Largemouth Bass (*Micropterus salmoides*). *Aquaculture Reports*.

[11] Wang K., Zhang L., Brown P. B. (2022). Effect of Replacement of Fish Meal With Cricket Meal on Growth Performance, Proximate Composition, Digestive Enzyme Activities, Serum Biochemical Indices, and Antioxidant Capacity in Largemouth Bass (*Micropterus salmoides*). *Aquaculture Research*.

[12] Su J., Liu Y., Xi L. (2022). The Effect of Dietary Tenebrio Monitor Meal Inclusion on Growth Performance and Liver Health of Largemouth Bass (*Micropterus salmoides*). *Journal of Insects as Food and Feed*.

[13] Sinkiewicz I., Śliwińska A., Staroszczyk H., Kołodziejska I. (2017). Alternative Methods of Preparation of Soluble Keratin From Chicken Feathers. *Waste and Biomass Valorization*.

[14] Verma A., Singh H., Anwar S. (2016). Microbial Keratinases: Industrial Enzymes With Waste Management Potential. *Critical Reviews in Biotechnology*.

[15] Maciel J. L., Werlang P. O., Daroit D. J., Brandelli A. (2017). Characterization of Protein-Rich Hydrolysates Produced Through Microbial Conversion of Waste Feathers. *Waste and Biomass Valorization*.

[16] Bhange K., Chaturvedi V., Bhatt R. (2016). Simultaneous Production of Detergent Stable Keratinolytic Protease, Amylase and Biosurfactant by Bacillus Subtilis PF1 Using Agro Industrial Waste. *Biotechnology Reports*.

[17] Paul T., Halder S. K., Das A. (2013). Exploitation of Chicken Feather Waste as a Plant Growth Promoting Agent Using Keratinase Producing Novel Isolate Paenibacillus Woosongensis TKB2. *Biocatalysis and Agricultural Biotechnology*.

[18] Hong C., Zhu J. Q., Zhao Y. M., Ma H. (2022). Effects of Dual-Frequency Slit Ultrasound on the Enzymolysis of High-Concentration Hydrolyzed Feather Meal: Biological Activities and Structural Characteristics of Hydrolysates. *Ultrasonics Sonochemistry*.

[19] Psofakis P., Karapanagiotidis I. T., Malandrakis E. E., Golomazou E., Exadactylos A., Mente E. (2020). Effect of Fishmeal Replacement by Hydrolyzed Feather Meal on Growth Performance, Proximate Composition, Digestive Enzyme Activity, Haematological Parameters and Growth-Related Gene Expression of Gilthead Seabream (*Sparus aurata*). *Aquaculture*.

[20] Fornari D. C., Nazeer S., Weldon A., Davis D. A. (2023). The Efficacy of Hydrolyzed Feathermeal as a Protein Source in Diets for Juvenile Catfish *Ictalurus punctatus*. *Aquaculture*.

[21] Yu R., Cao H., Huang Y. (2019). The Effects of Partial Replacement of Fishmeal Protein by Hydrolysed Feather Meal Protein in the Diet With High Inclusion of Plant Protein on Growth Performance, Fillet Quality and Physiological Parameters of Pengze Crucian Carp (*Carassius auratus* var. Pengze). *Aquaculture Research*.

[22] Horwitz W. (1970). *Official Methods of Analysis of the Association of Official Analytical Chemists*.

[23] Yang P., Wang W., Chi S., Mai K., Song F., Wang L. (2020). Effects of Dietary Lysine on Regulating GH-IGF System, Intermediate Metabolism and Immune Response in Largemouth Bass (*Micropterus salmoides*). *Aquaculture Reports*.

[24] Gu J., Liang H., Ge X. (2022). A Study of the Potential Effect of Yellow Mealworm (*Tenebrio Mollitor*) Substitution for Fish Meal on Growth, Immune and Antioxidant Capacity in Juvenile Largemouth Bass (*Micropterus salmoides*). *Fish & Shellfish Immunology*.

[25] Zhao L., Liang J., Chen F. (2021). High Carbohydrate Diet Induced Endoplasmic Reticulum Stress and Oxidative Stress, Promoted Inflammation and Apoptosis, Impaired Intestinal Barrier of Juvenile Largemouth Bass (*Micropterus salmoides*). *Fish and Shellfish Immunology*.

[26] Zhao X., Li L., Li C., Liu E., Zhu H., Ling Q. (2022). Heat Stress-Induced Endoplasmic Reticulum Stress Promotes Liver Apoptosis in Largemouth Bass (*Micropterus salmoides*). *Aquaculture*.

[27] Li P., Wu G. (2018). Roles of Dietary Glycine, Proline, and Hydroxyproline in Collagen Synthesis and Animal Growth. *Amino Acids*.

[28] He Z., Sun R., Tang Z. (2018). Biodegradation of Feather Waste Keratin by the Keratin-Degrading Strain Bacillus Subtilis 8. *Journal of Microbiology and Biotechnology*.

[29] Campos I., Matos E., Marques A., Valente L. M. P. (2017). Hydrolyzed Feather Meal as a Partial Fishmeal Replacement in Diets for European Seabass (*Dicentrarchus labrax*) Juveniles. *Aquaculture*.

[30] Badillo-Zapata D., Vega-Villasante F., Nolasco-Soria H., López-Acuña L., Vargas-Ceballos M. A., Barreto-Curiel F. (2023). Replacement of Fishmeal by Hydrolyzed Feather Meal in Diets of Juvenile *Macrobrachium tenellum* (River Prawns) and Its Effect on Muscle Fatty Acids. *Latin American Journal of Aquatic Research*.

[31] Wilfart A., Espagnol S., Dauguet S., Tailleur A., Gac A., Garcia-Launay F. (2016). ECOALIM: A Dataset of Environmental Impacts of Feed Ingredients Used in French Animal Production. *PLoS ONE*.

[32] Fowler L. G. (1990). Feather Meal as a Dietary Protein Source During Parr-Smolt Transformation in Fall Chinook Salmon. *Aquaculture*.

[33] Fasakin E. A., Serwata R. D., Davies S. J. (2005). Comparative Utilization of Rendered Animal Derived Products With or Without Composite Mixture of Soybean Meal in Hybrid Tilapia (*Oreochromis niloticus×Oreochromis mossambicus*) Diets. *Aquaculture*.

[34] Li K., Wang Y., Zheng Z-Xing, Jiang R-Li, Xie N-Xia, Bureau D. P. (2009). Replacing Fish Meal With Rendered Animal Protein Ingredients in Diets for Malabar Grouper, *Epinephelus malabaricus*, Reared in Net Pens. *Journal of the World Aquaculture Society*.

[35] Zhang Q., Liang H., Longshaw M. (2022). Effects of Replacing Fishmeal With Methanotroph (*Methylococcus capsulatus*, Bath) Bacteria Meal (FeedKind) on Growth and Intestinal Health Status of Juvenile Largemouth Bass (*Micropterus salmoides*). *Fish & Shellfish Immunology*.

[36] Hartviksen M., Bakke A. M., Vecino J. G., Ringo E., Krogdahl A. (2014). Evaluation of the Effect of Commercially Available Plant and Animal Protein Sources in Diets for Atlantic Salmon (*Salmo salar L*.): Digestive and Metabolic Investigations. *Fish Physiology and Biochemistry*.

[37] Oliva-Teles A. (2012). Nutrition and Health of Aquaculture Fish. *Journal of Fish Diseases*.

[38] Amir Aslani B., Ghobadi S. (2016). Studies on Oxidants and Antioxidants With a Brief Glance at Their Relevance to the Immune System. *Life Sciences*.

[39] Yu X., Wu Z., Fu Y. (2024). Replacement of Dietary Fish Meal With Soy Protein Concentrate on the Growth Performance, PI3K/AKT/TOR Pathway, Immunity of Abalone Haliotis Discus Hannai and Its Resistance to Vibrio Parahaemolyticus. *Aquaculture Reports*.

[40] Dragun Z., Marijić V. Filipović, Krasnići N. (2017). Malondialdehyde Concentrations in the Intestine and Gills of Vardar Chub (*Squalius vardarensis Karaman*) as Indicator of Lipid Peroxidation. *Environmental Science and Pollution Research*.

[41] Chen S., Zeng J., Li R. (2024). Traditional Chinese Medicine in Regulating Macrophage Polarization in Immune Response of Inflammatory Diseases. *Journal of Ethnopharmacology*.

[42] Wang S., Cai M., Wang Y., Zhong L., Hu Y., Fu G. (2024). Dietary Clostridium Butyricum Metabolites Mitigated the Disturbances in Growth, Immune Response and Gut Health Status of Ctenopharyngodon Idella Subjected to High Cottonseed and Rapeseed Meal Diet. *Fish and Shellfish Immunology*.

[43] Yang M., Wang Y., Patel G. (2020). In Vitro and in Vivo Anti-Inflammatory Effects of Different Extracts From Epigynum Auritum Through Down-Regulation of NF-*κ*B and MAPK Signaling Pathways. *Journal of Ethnopharmacology*.

[44] Li T., Zhou X. Q., Jiang W. D. (2017). Sodium Butyrate Improved Intestinal Immune Function Associated With NF-*κ*B and p38MAPK Signalling Pathways in Young Grass Carp (*Ctenopharyngodon idella*). *Fish and Shellfish Immunology*.

[45] Wang T., Secombes C. J. (2013). The Cytokine Networks of Adaptive Immunity in Fish. *Fish and Shellfish Immunology*.

[46] Roh H., Park J., Kim A. (2020). Overfeeding-Induced Obesity Could Cause Potential Immuno-Physiological Disorders in Rainbow Trout (*Oncorhynchus mykiss*). *Animals*.

[47] Peng Y., Xie S., Liu Z. (2019). Effects of Dietary Oxidized Fish Oil on Growth Performance, Antioxidant Defense System, Apoptosis and Mitochondrial Function of Juvenile Largemouth Bass (*Micropterus salmoides*). *Aquaculture*.

[48] Chen X., Bian M., Zhang C. (2018). Dihydroartemisinin Inhibits ER Stress-Mediated Mitochondrial Pathway to Attenuate Hepatocyte Lipoapoptosis via Blocking the Activation of the PI3K/Akt Pathway. *Biomedicine and Pharmacotherapy*.

[49] Yang Y., Li C., Liu N. (2021). Ursolic Acid Alleviates Heat Stress-Induced Lung Injury by Regulating Endoplasmic Reticulum Stress Signaling in Mice. *Journal of Nutritional Biochemistry*.

[50] Zhao X., Mao W., Lin Z., Ling Q. (2024). Heat Stress Induced Hepatocyte Apoptosis in Largemouth Bass *Micropterus salmoides* via IRE1*α*/TRAF2/ASK1/JNK Pathway. *Journal of Oceanology and Limnology*.

